# Spectrum of *ctx*B genotypes, antibiogram profiles and virulence genes of *Vibrio cholerae* serogroups isolated from environmental water sources from Odisha, India

**DOI:** 10.1186/s12866-023-02811-2

**Published:** 2023-03-16

**Authors:** Bibhuti Bhusan Pal, Debasish Samal, Smruti Ranjan Nayak, Swatishree Pany

**Affiliations:** Microbiology Division, ICMR-Regional Medical Research Centre, 751023 Chandrasekharpur, Bhubaneswar, Odisha India

**Keywords:** Genomic diversity, Clonality, *V. cholerae* O1, Water isolates, Odisha

## Abstract

**Background:**

The present study reports on the comprehensive analysis of *Vibrio cholerae* O1 and non-O1/non-O139 serogroups isolated from environmental water sources during cholera outbreaks, epidemics and surveillance studies between years 2007 to 2019 from different districts of Odisha, India.

**Methods:**

A total of 85 stocked cultures of *V. cholerae* O1 and non-O1/non-O139 strains were analyzed for different *ctx*B genotypes, toxic genes, antibiogram profiles through PCR assays and pulsotyped by pulsed-field gel electrophoresis (PFGE).

**Results:**

From all *V. cholerae* strains tested, 51 isolates were O1 Ogawa and the rest 34 strains were non-O1/non-O139. All the *V. cholerae* O1 strains were altered El Tor variants carrying *ctx*B1, *ctx*B3 and *ctx*B7 genotypes. However, only *ctx*B1 genotypes were present in *V. cholerae* non-O1/non-O139. Though non-O1/non-O139 strains were negative by O1 antisera, 20% strains were positive for *rfbO1* gene by PCR assay. All the *V. cholerae* isolates possessed a variety of virulence genes including *ace, ctxAB, toxR, zot, hlyA* which were in higher percentage in the case of *V. cholerae* O1. The *Vibrio cholerae* O1 and non-O1-/non-O139 strains showed multiple antibiotic resistances in 2007 and 2012. The PCR detection of four resistance associated genes (*strB, dfrA1, sulll, SXT*) confirmed higher prevalence in *V. cholerae* non-O1/non-O139 strains. The PFGE analysis revealed 3 pulsotypes having 93% similarity among *V. cholerae* O1 strains.

**Conclusion:**

This study indicates the changing epidemiology, antibiogram patterns and continuous genetic variation in environmental *V. cholerae* strains of Odisha over the years. So continuous surveillance is necessary to understand the changing patterns of *V. cholerae* different serogroups isolated from stool and water samples from Odisha.

## Introduction

*Vibrio cholerae* is an etiological agent of cholera which causes life-threatening severe diarrhea worldwide. According to the reports published by WHO, an estimated 3–5 million cholera cases are annually reported worldwide [1]. Out of more than 200 serogroups identified on the variation in O-antigenic lipo-polysaccharide (LPS), only *V. cholerae* O1 and O139 caused outbreaks/epidemics of cholera worldwide [2]. Historically, the *V. cholerae* O1 serogroups have two major biotypes, the classical and El Tor, which differ in phenotypic and genotypic traits [3]. The *V. cholerae* O1 classical biotype, which would have caused the first six pandemics of cholera [4] was replaced by the El Tor biotype, which is responsible for the ongoing seventh pandemic since 1961. Again, El Tor biotype strains have undergone genetic mutation to evolve as hybrid El Tor carrying classical *ctx*B of classical biotype [5]. The scientific literature suggests that *V. cholerae* transmits through fecal-oral routes by contaminated water [6]. It has also been reported that these *V. cholerae* strains became more pathogenic after human-to-human transmission through fecal-oral route [7]. Therefore, it has been proved that both the cholera patients and environmental reservoirs can be considered as potential sources of outbreaks. However, the source and mode of transmission for the amplification of the disease to reach an epidemic form is still not clearly understood.

**Fig. 1:**
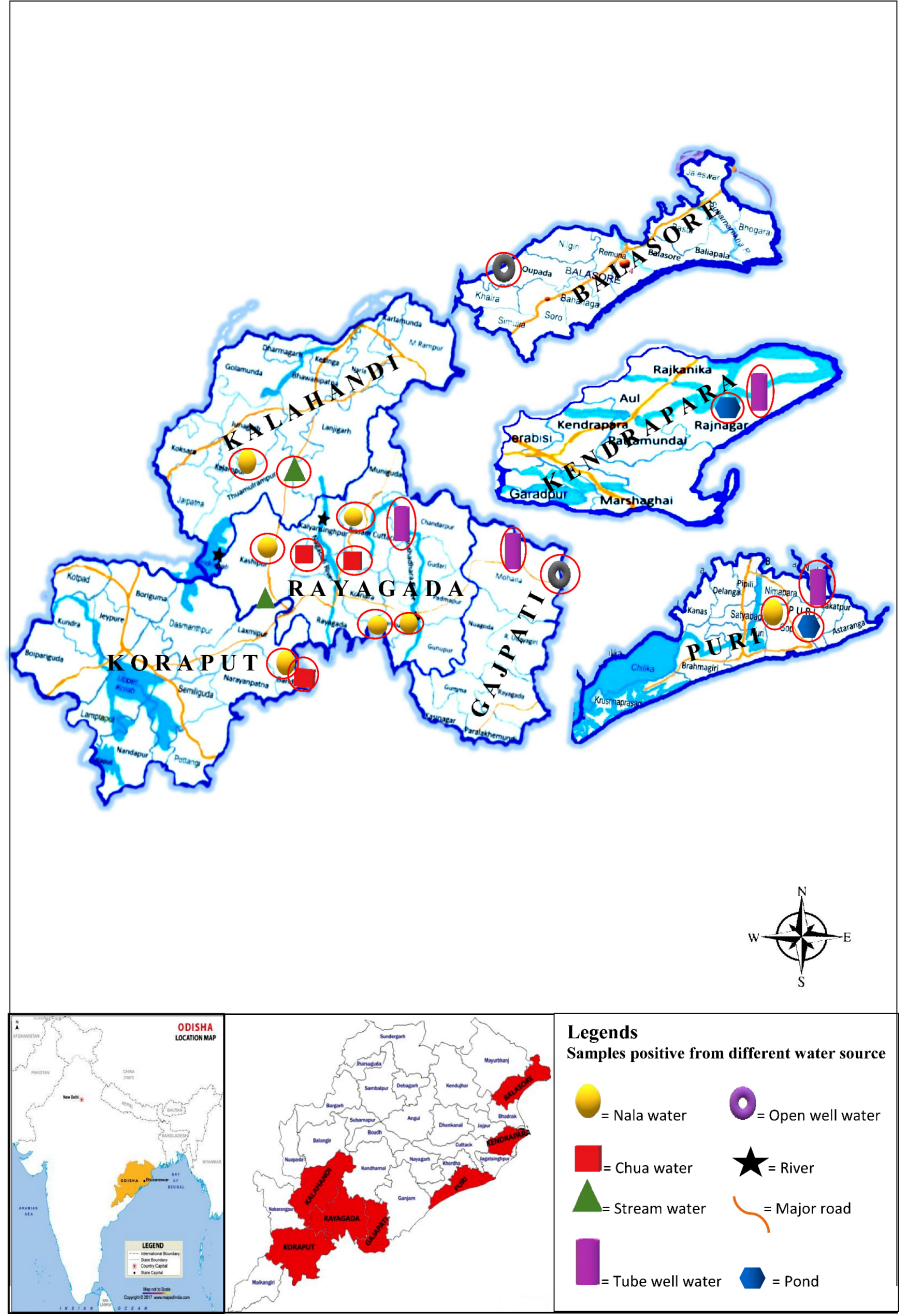
Map showing the distribution of water sources positive for *Vibrio cholerae* O1 and non-O1/non-O139 strains

Odisha is an eastern state of India located along the Bay of Bengal between 17.780 and 22.730 N latitudes and 81.370 E to 87.530 E longitudes. It is comprised of 30 administrative districts with total land area of 155,707 sq km (Fig. 1). The total population of the state is about 42 million (scheduled tribe: 22.85%, scheduled caste:17.13%) and 83.3% of them live in rural areas as per census 2011. The western and southern districts are the hilly and forest areas inhabited by tribal population in inaccessible areas and they live in unhygienic living conditions; whereas the coastal eastern districts have been experiencing natural calamities like flood and cyclone almost every year. Odisha has experienced so many cholera outbreaks and epidemics since 1993 (epidemics in tribal areas), 1999 (after super cyclone in coastal districts), 2007 (cholera epidemic in tribal areas), and again in 2012, 2014, 2016 and 2019 both in the coastal and tribal areas, as it was reported in our previous studies [8–11]. Cholera has become endemic in Puri district (coastal district) whereas seasonal in tribal areas. During these three decades, numerous epidemiological and molecular studies have been carried out in our laboratory. The environmental reservoirs were studied in both coastal and tribal areas of Odisha [12]. However, there is no in-depth studies conducted on the environmental isolates of *V. cholerae* serogroups that were isolated from both the coastal and tribal areas of Odisha. Thus, the present study focused on the detailed molecular analysis of different toxic genes, antibiotic resistance genes, antibiogram profiles and PFGE of both O1 and non-O1/non-O139 *V. cholerae*, which were isolated from environmental water sources in different coastal and tribal areas of Odisha and preserved in the laboratory.

## Materials and methods

### Revival of strains

A total of 85 environmental strains of *V. cholerae* O1 and non-O1/non-O139 from laboratory stocks were included in this study. These strains were isolated between 2007 and 2019 from the coastal and tribal areas of Odisha during cholera outbreaks/epidemics and surveillance studies (Fig. 1). Laboratory stocked *V. cholerae* strains isolated from environmental water samples were revived and confirmed using standard laboratory protocols [13, 14]. Selected colonies from Thiosulfate-Citrate-Bile salts-Sucrose (TCBS) agar plates were re-streaked onto Luria Bertani (LB) agar plates (BD, USA), further selected colonies were inoculated in Luria Bertani broth (BD, USA) to carry out genomic DNA extraction using boiling method [10]. Before this, the serology of the *V. cholerae* isolates was confirmed by slide agglutination test using specific polyvalent O1 and O139 antisera and mono-specific Ogawa and Inaba antisera (BD, USA). The detailed molecular analysis of *V. cholerae* O139 strains were published on another publication [15]. Presently, all the *V. cholerae* O1 and non-O1/non-O139 strains are analyzed in this communication.

### PCR assays

All the revived strains which were phenotypically identified as *V. cholerae* were further reconfirmed by performing simplex PCR targeting the species-specific gene *ompW* and identifying gene *rfb*O1. The presence of major virulence and regulatory genes of *V. cholerae i.e. tcpA, ace, rtxC, ompU, toxR, ctxAB, hlyA* and *zot* were screened through multiplex PCR assays [16]. The amplification of the antibiotic resistance genes *dfrA1, sulll, SXT* and *strB* were also carried out through a multiplex PCR assay by simultaneous addition of all primer pairs in the same reaction mixture [17]. The amplified PCR products were separated on 1.8% agarose gel stained with ethidium bromide using gel electrophoresis technique and were visualized under gel documentation system (Bio-Rad, USA).

### Mismatch amplification mutation assay (MAMA) and double mismatch amplification mutation assay (DMAMA) PCR

MAMA and DMAMA PCR assays were performed to distinguish between classical (*ctxB1*), El Tor (*ctx*B3) and haitian (*ctx*B7) genotypes in all *V. cholerae* isolates from 2007 to 2019 using biotype specific primers [18, 19].

### Antibiotic susceptibility assay

The antibiotic susceptibility patterns of *V. cholerae* O1 and non-O1/non-O139 isolates were identified using the Kirby-Bauer disc diffusion method on Muller Hinton Agar (MHA) plates as described by the Clinical and Laboratory Standard Institute [20] and from our previous laboratory practices [21]. A total of 14 commercially available antibiotic discs used for this assay were as follows: - ciprofloxacin (Cf), gentamicin (G), azithromycin (At), ampicillin (A), chloramphenicol (C), tetracycline (T), streptomycin (S), nalidixic acid (Na), neomycin (N), doxycycline (Do), ofloxacin (Of), norfloxacin (Nx), cotrimoxazole (Co), and furazolidone (Fr) (BD, USA). The zone of inhibition was measured to determine the resistance profiles of *V. cholerae* isolates.

### Pulsotyping by pulsed-field gel electrophoresis (PFGE)

A total of 21 randomly selected *V. cholerae* O1 strains isolated from 2007 to 2019 were pulsotyped by PFGE technique in a CHEF Mapper system (Bio-Rad, USA), according to the procedures described elsewhere [22]. The genomic DNA of *V. cholerae* in agarose plugs were restricted with 50U of Not I (New England Biolabs, USA). The Not I digested restriction DNA fragments were separated through 1% pulse-field certified agarose gel (Bio-Rad, USA) in 0.5X TBE buffer. The DNA was visualized after staining and destaining of the gel and the images were digitized by gel documentation system (Bio-Rad). The fingerprint patterns in gel were analyzed using BioNumerics software V7.6.

## Results

### Study area

This study was conducted from previously isolated *V. cholerae* O1 and non-O1/non-O139 strains which were isolated from environmental water sources in coastal and tribal districts during cholera outbreaks, epidemics and surveillance studies between 2007 and 2019 from Odisha, India (Fig. 1). These strains were from the coastal districts of Puri, Kendrapara, Balasore, and from the tribal districts of Rayagada, Koraput, Kalahandi, Gajapati, which were the most cholera-affected districts in Odisha as reported earlier. These strains were isolated from major rivers in southern Odisha such as Nagavali and Kalyani, which flows through the routinely cholera-affected tribal districts of Kalahandi, Rayagada and Koraput. Samples from streams, open wells, tube wells, nala (narrow water stream) and chua (small water reserve in river bed) were collected from sites easily accessible to people where cases were reported. A map showing the spread of *V. cholerae* O1 strains isolated from different water sources during cholera outbreaks/epidemics and surveillance studies in coastal and tribal regions of Odisha was prepared (Figure-1).

#### Serological identification and typing of V. cholerae

A total of 85 revived strains produced characteristic moist yellow colonies of *V. cholerae* on the selective TCBS medium, out of which 51 strains were confirmed as *V. cholerae* O1 Ogawa and the remaining 34 strains were confirmed as *V. cholerae* non-O1/non-O139 when tested in sero-specific antisera (BD, USA).

#### Prevalence of virulence associated genes (VAGs)

**Fig. 2:**
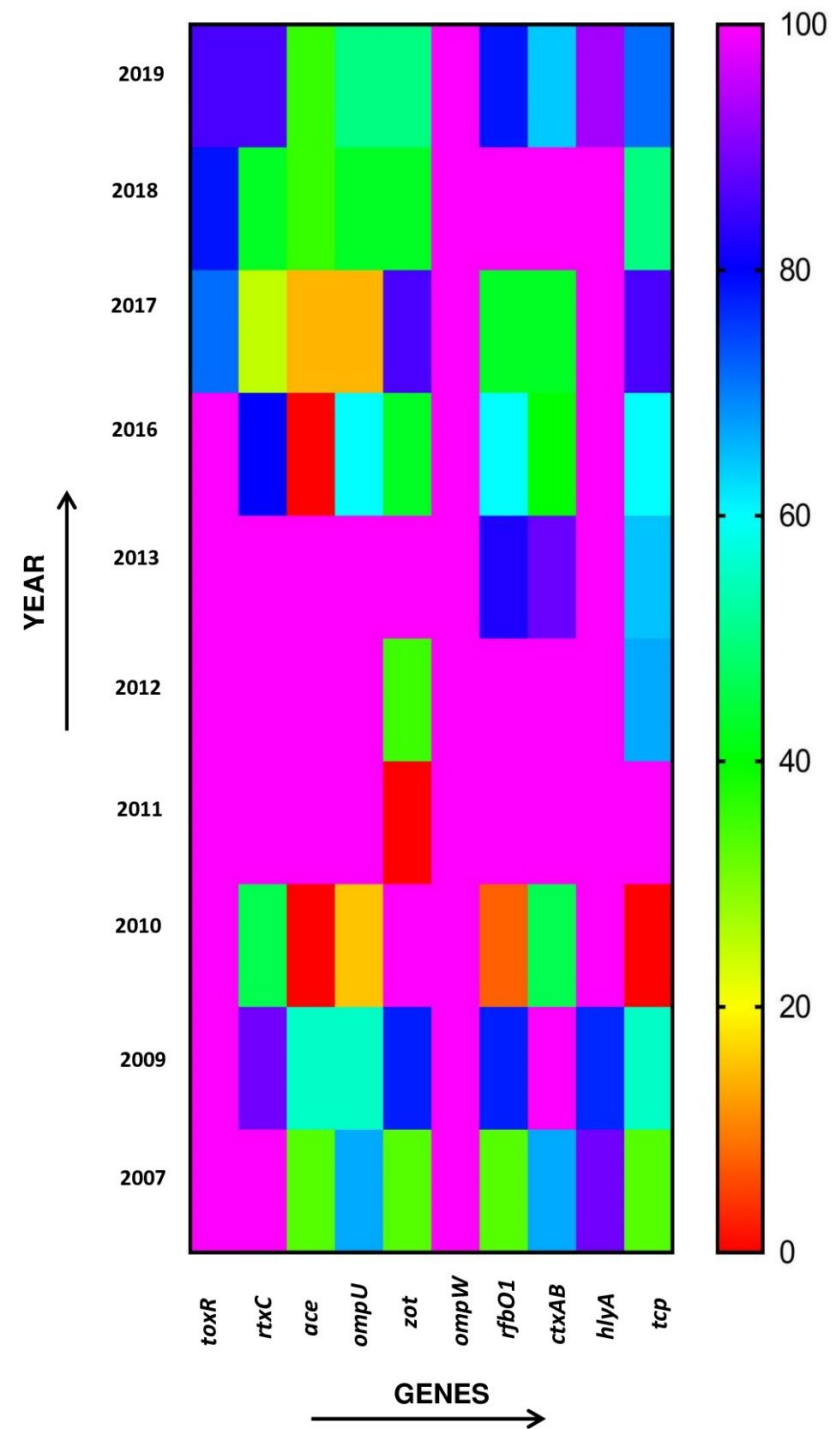
Heat map showing percentage variation of different virulence genes among *Vibrio cholerae* O1 and non-O1/non-O139 strains over the years

The *V. cholerae* isolates were screened to obtain the virulence-associated gene profiles through the multiplex PCR assays. All 85 *V. cholerae* O1 and non-O1/non-O139 were positive for *ompW* gene and 51 of them which were serologically confirmed as *V. cholerae* O1 also possessed *rfb*O1 gene, whereas in the remaining 34 isolates almost all failed to amplify the ‘O1’ serogroup specific gene. Thus, it completely validated the serological results obtained. All *V. cholerae* isolates were positive for El Tor biotype *tcp*A Haitian variant and showed complete dominance over *tcp*A classical and *tcp*A El Tor from 2007 to 2019. Almost all *V. cholerae* O1 strains were positive for the following virulence and regulatory genes: the cholera toxin transcriptional activator *toxR* (84.3%), the repeat in toxin *rtxC* (78.4%), the accessory cholera enterotoxin *ace* (64.7%), the zonula occuludens toxin *zot* (52.9%), the outer membrane protein *ompU* (68.6%), toxin corregulated pilus *tcp* (68.6%), haemolysin *hlyA* (88.25%) and cholera toxin *ctxAB* (92.2%). In case of non-O1/non-O139 *V. cholerae* strains carrying virulence and regulatory genes were 94% positive for *toxR*, 60% *rtxC*, 14% *ace*, 34% *ompU*, 38.2% *zot*, 85.29% *hlyA*, 29.41% *tcp* and 47% *ctxAB*. However, 20% of the *V. cholerae* non-O1/non-O139 isolates were positive for *rfb*O1 gene which were negative by serology (Table-1). The annual prevalence of the virulence associated genes in *V. cholerae* O1 is represented in a heat map (Figure-2).

### MAMA and D-MAMA PCR assays

These assays exhibited that environmental *V. cholerae* O1 strains carried the *ctx*B7 genotype, which were abundant from 2007 to 2019; but became quiescent in 2010 to 2011, where *ctx*B3 genotype were predominant within different districts of Odisha. Although *ctx*B1 and *ctx*B7 genotypes coexisted in 2012 and 2013, but thereafter *ctx*B1 classical genotype was completely replaced by *ctx*B7 Haitian genotype in the environmental strains isolated from both the coastal and tribal areas of Odisha. In contrast, 50% of *V. cholerae* non-O1/non-O139 isolates were positive for the classical (CT) *ctx*B1 genotype and the rest of them failed to amplify the primer binding region of all the three *ctx*B genotypes. The distribution of *ctx*B genotypes from 2007 to 2019 in *V. cholerae* O1 and non-O1/non-O139 strains is represented in Figure- 3a and 3b respectively.

### Profiling of antibiotic resistant genes

While testing the antibiotic resistance genes by PCR assay, it showed positive results when amplified a 278 bp fragment of *dfrA1*, a 515 bp of *strB*, a 626 bp of *sulll* and a 1035 bp of *SXT* element. The *V. cholerae* strains possessing all of these genes might have conferred resistance against ampicillin, nalidixic acid, streptomycin, furazolidone, cotrimoxazole and neomycin. Most of the *V. cholerae* O1 strains carried antibiotic resistant genes, with 94% being positive for *dfrA1*, 80% for *SXT*, 57% for *sulll* and 51% for *strB*. However, *V. cholerae* non-O1/non-O139 showed different prevalence of antibiotic resistance genes than in *V. cholerae* O1 with 88% being positive for *dfrA1*, 76% positive for *SXT*, 67% positive for both *sulll* and *strB*. The distribution profiles of these drug resistant genes in *V. cholerae* strains were represented in Figure-4.

### Antibiotic resistance patterns and multiple antibiotic resistance (MAR) index

Antimicrobial susceptibility test for 85 environmental *V. cholerae* isolates against 14 different antibiotics in this setting was carried out by disc diffusion method. All the tested *V. cholerae* isolates were uniformly sensitive to norfloxacin, and doxycycline. In addition, 90% of the *V. cholerae* isolates were susceptible to both ofloxacin and tetracycline; whereas the rest 10% of them exhibited reduced susceptibility in 2010 and 2011 which was later reversed. All the *V. cholerae* O1 isolates were mainly resistant against nine commonly used antibiotics; whereas *V. cholerae* non-O1/non-O139 were resistant to 5 different antibiotics. Thus, the antibiotic sensitivity profiles of the *V. cholerae* isolates represents that non-O1/non-O139 have higher susceptibility to tested antibiotics in comparison to *V. cholerae* O1 isolates (Table-2). The *V. cholerae* O1 strains produced a MAR index value of at-least 0.64 in all strains tested represented in box whisker plot (Figure-5), thus they all were confirmed as MDR strains. The antibiotic resistance patterns among *V. cholerae* O1 and non-O1/non-O139 strains displayed a brisk variation over past few years. The resistance of *V. cholerae* O1 against gentamicin, ciprofloxacin and chloramphenicol declined from 90% to 2007 to almost zero by 2019. However, the resistance against azithromycin and cotrimoxazole fluctuated between 33 and 100% in *V. cholerae* O1 during this period which is represented in (Figure-6). Overall, 51% of all the isolated *V. cholerae* strains between 2007 and 2019 were resistant to 7 or more different antibiotics.

### PFGE analysis

The PFGE banding patterns of the *Not*-I digested genomic DNA of 11 representative *V. cholerae* O1 strains isolated from environmental water sources from different districts of Odisha exhibited 3 different pulsotypes having 93% similarity (Figure-7).

## Discussion

Cholera still continues as a major public health menace for centuries in developing and underdeveloped countries of Asia and Africa. The abrupt and deadly onset of cholera epidemics and outbreaks have been a persistent health problem in coastal and tribal regions of Odisha, India [9]. This has been exacerbated by unhygienic living practices, inadequate health infrastructures and limited access to potable drinking water. The consumption and utilization of contaminated water from environmental sources like streams, nalas and rivers made the population prone to cholera outbreaks and epidemics in rainy seasons, such as the major outbreaks of 2007 and 2010 in Rayagada district, of 2009 in Kendrapara district and 2014 in Kalahandi district of Odisha [10, 23]. In the mid of 2007, a severe cholera epidemic was reported in the kashipur block of Rayagada district and continued transmitting to the neighboring districts such as Gajapati, Koraput, and Kalahandi leading to be one of the most extreme cholera outbreaks in Odisha [14].

This study provides a comprehensive analysis of the diversities of virulence genes, antibiotic resistance genes and antibiogram trend over more than a decade from 2007 to 2019 along with the co-existence of *V. cholerae* O1 and non-O1/non-O139 strains from environmental sources like nala, chua, streams, ponds, rivers and road side reservoirs. The results from this study confirmed higher prevalence of *V. cholerae* O1 than non-O1/non-O139 in environmental water samples which contradicts to the result reported in Bepanda, Douala [24]. At present, the altered El Tor strains of *V. cholerae* O1 carrying *ctx*B7 genotypes were identified in all the environmental isolates from 2007 onwards and it predominated over the normal prototype El Tor strains causing all outbreaks and epidemics in Odisha. Our earlier studies proved that these strains carrying *ctx*B7 genotype was first reported from the coastal district of Odisha during super cyclone of 1999 [10]. Our data also supported the fact that environmental and clinical El Tor strains having *ctx*B7 gene were simultaneously circulating in the tribal districts of Odisha subsequent to the cholera epidemic in 2007 [12]. However, by correlating with the results from our earlier studies on clinical strains it was evident that both clinical and environmental strains showed 100% prevalence of *tcp*A Haitian genes from 2007 to 2019 except in years 2011 to 2013 and 2016 where clinical strains fall behind [21].

The multiplex PCR-I established the presence of different genes like *zot, tcpA, rfbO1, ctxAB, and ompW* genes; whereas multiplex PCR-II analysis confirmed the presence of *toxR, ompU, hlyA, ace*, and *rtxC* genes. The serological result complemented with the results of multiplex PCR I assay. This assay confirmed that all the 51 *V. cholerae* strains were toxigenic as they were positive for *ompW, rfb*O1 and *ctxAB* genes [16]. However, 20% of the 34 *V. cholerae* non-O1/non-O139 strains that were serologically negative possessed sero-specific *rfb*O1 gene confirmed through multiple PCR-I assay, which was an exception. The multiplex PCR-II assays possessing the biotype-specific marker genes like *rtxC, tcpA* and *ctxB* revealed that the confirmed *V. cholerae* O1 were of altered El Tor prototype but mostly possessed Haitian *tcp*A and *ctx*B Haitian genotypes. However, the results produced were contradictory to the findings reported in Bepanda in Doula, Accra in Ghana, and Dhaka in Bangladesh, as the prevalence of *tcp*A El Tor and *ctx*B1 genotypes were more prominent in these regions [24–26]. Generally, the environmental isolates of *V. cholerae* non-O1/non-O139 strains were incompetent to seed any cholera outbreaks/epidemics; but the seroconversion of *V. cholerae* non-O1/non-O139 to O1 has heightened attention towards *V. cholerae* non-O1/non-O139 strains. Though non-O1/non-O139 serogroup of *V. cholerae* are non-lethal, evidence of genes encoding virulence factors have been identified in environmental strains, which might account for major public health risks [27].

From the present study, it was evident that the prevalence of essential toxic genes such as *toxR, zot* and *ace* of *V. cholerae* O1 strains were 50% and above, along with the accessory genes i.e. *rtxC*, *ompU*, *ctxAB*, *hlyA*, *ompW*, *rfb*O1 and *tcpA*, which varied in-between 68.6 and 100%. These findings resembled those reported by other researchers from Accra, Ghana in 2019, Bangladesh in 2004 and Lubumbashi, Congo in 2008 [25, 28, 29]. From all the virulence and accessory genes in *V. cholerae* non-O1/nonO139 tested, only 3 genes were prominent i.e. *toxR* (94.3%), *rtxC* (60%), and *hlyA* (85.7%). Although *V. cholerae* non-O1/non-O139 strains typically do not possess virulence associated genes, they often contain other virulence genes, which can contribute to their pathogenicity. It has also been reported that both *V. cholerae* O1 and non-O1/non-O139 isolated from the same aquatic habitat possessed the regulatory protein gene (*toxR*). Thus it can be speculated that under several biochemical and physiological conditions, horizontal gene transfer might have occurred from toxigenic *V. cholerae* O1 to non-O1/non-O139 when exposed to bacteriophages. This could lead to the emergence of novel pathogenic *V. cholerae* strains in the environment causing severe outbreaks/epidemics [30].

The genetic basis for the resistance to antibiotics could be due to lateral acquisition of self-transmissible conjugative SXT element in *V. cholerae* derived ICE element. The name SXT was given because of its contribution to help bacteria to sustain and grow in the presence of streptomycin, sulfamethoxazole and trimethoprim. SXT ICEs possibly emerged first from the Asian continents during the late 1980s and subsequently spread into several clinically important bacterial species in different countries. Currently, SXT ICEs were detected in almost every *V. cholerae* O1 isolates across the globe. Genomic studies indicated that *V. cholerae* O1 and O139 isolates independently acquired the SXT ICEs. [31, 32]. The ICE-associated genes *dfrA1*, *sulII*, *SXT* element and *strB* were reported as the genetic determinant to carry resistance against multiple drugs such as streptomycin, cotrimoxazole, nalidixic acid and neomycin [33]. In the present study, we focused on the ICE-associated genes *SXT, strB, dfrA1* and *sulll*, which conferred resistance to different serogroups of *V. cholerae* against multiple drugs. The resistance genes *dfrA1* and *SXT* were mostly prevalent in 94% and 80% of the *V. cholerae* O1 isolates respectively, in addition, the *Sulll and strB* genes were also prevalent in 50% and 57% of the strains. *V. cholerae* harboring these multiple antibiotic markers in the genome displayed resistance against ampicillin, streptomycin, nalidixic acid, neomycin, cotrimoxazole, and furazolidone [34]. The vast spread of *dfrA1* and *SXT* elements in the strains tested resulted in the resistance of these antibiotics. Besides all, the mobile integron often linked with the MGEs (ICE, insertion sequence, transposons, conjugative plasmids) is also responsible for the dissemination of multiple antibiotic resistance encoding functions to the environmental *V. cholerae* strains. However, MGEs were the pre-existing source of antimicrobial-resistant genes which mediated the exchange of antibiotic resistance genes between pathogens or between pathogens and non-toxigenic commensals of bacterial populations which were existing in common environmental niches [32]. Thus mobile integrons, ICE, and conjugative plasmids could also attribute the genetic basis for multi-drug resistance in *V. cholerae* strains.

Effective treatment in cholera cases can be only possible through aggressive oral or intravenous re-hydration therapy along with the ideal course of antibiotics. This can significantly shorten the duration of diarrhea and reduce the loss of electrolytes in the patients’ body [3]. However, difficulties in antibiotic therapy have evolved in recent years due to the rapid emergence of multi-drug resistant (MDR) strains of *V. cholerae* in different serogroups from Asia, Africa, and America [35]. Over the past two decades, progressive increasing trend of *V. cholerae* O1 resistance against multiple drugs such as tetracycline, streptomycin, norfloxacin, ciprofloxacin, trimethoprim/sulfamethoxazole, ampicillin, neomycin, cotrimoxazole, nalidixic acid, and furazolidone were reported in Odisha and India [21]. The antibiotic susceptibility assay revealed loss of sensitivity to the tested antibiotics including nalidixic acid, streptomycin, co-trimoxazole, furazolidone, ampicillin and neomycin which were used in the first line of defense against cholera. Similar incidences of resistance patterns were also reported in Nepal in 2015, Ghana in 2019, and the Democratic Republic of Congo in 2015 [25, 36, 37]. In the present study, both *V. cholerae* O1 and non-O1/non-O139 were completely resistant to ampicillin, furazolidone, and streptomycin. A higher percentage of resistance from 60 to 80% against azithromycin, cotrimoxazole, nalidixic acid and neomycin in *V. cholerae* O1 strains were observed since 2007. These results coincided with the reports from Nepal and Mozambique [38, 39]. Tetracycline-resistant *V. cholerae* O1 in clinical strains were reported only in 2010 from tribal areas of Odisha, whereas in the present study on environmental isolates both *V. cholerae* O1 and non-O1/non-O139 strains were resistant to tetracycline on year 2010 and 2011. However, it was reversed in successive years [40]. The environmental isolates of *V. cholerae* during cholera epidemics and outbreaks were observed having higher antibiotic resistances and this might me due to the transmission of multi-drug resistant *V. cholerae* O1 strains from human to water bodies. The drug resistant patterns of both clinical and environmental strains varied over space and time.

In this study, it was discovered that 66.6% of all the *V. cholerae* O1 isolates were resistant to at least 7 out of 14 antibiotics screened and possessed a MAR index value ranging between 0.5 and 0.79. However, 73.5% of the non-O1/non-O139 were sensitive to more than 8 antibiotics screened. The highest MAR values were reported in 2007 and 2012; as 10 out of 14 antibiotics were resistant to *V. cholerae* O1 environmental water isolates. This could be due to prolonged usage of antibiotics for curative and prophylactic purposes in cholera-affected patients during the outbreaks and epidemics occurred in the Rayagada, Gajapati and Kalahandi districts of Odisha. This might have provoked the emergence and spread of MDR *V*. *cholerae* strains in the environment. However, the reversal in the resistance of these antibiotics against *V. cholerae* was also observed in successive years. This phenomenon might be due to rapid fluctuation in nature and the inability of *V. cholerae* to carry plasmids that conferred resistance to it [41].

*NotI* digested genomic DNA of *V. cholerae* O1 from water isolates were analyzed by PFGE, whose patterns revealed diversity of their genomes containing 3 different pulsotypes with a similarity matrix of 93%. The PFGE banding patterns in 8 strains were highly homogenous showing 100% similarity indicating origin from a single clone. Whereas, the remaining 3 pulsotypes of 2012 and 2018 possessing *ctx*B1 and *ctx*B7 genotypes respectively were distinct from the other strains. The present study also indicates that the environmental isolates of *V. cholerae* were the precursors of all the outbreaks and epidemics of cholera in Odisha [26].

Thus, continuous surveillance is necessary to monitor the different water bodies to study the changing over of biotypes and serotypes of *V. cholerae* different serogroups which will give early warning system for the control of cholera outbreaks/epidemics in this area. Again, in-depth molecular study on the toxic genes, drug resistance patterns including the *ctx*B genotypes of *V. cholerae* strains from coastal and tribal areas of Odisha is highly essential.

**Table 1:** Prevalence of virulence genes in *Vibrio cholerae* O1 and non-O1/non-O139 strains isolated from environmental sources in different districts of Odisha: 2007–2019

Serial No.	YEAR	AREA	SEROGROUP (n)	*tox*R (%)	*rtx*C (%)	*ace* (%)	*omp*U (%)	*zot* (%)	*omp*W (%)	*rfb*O1 (%)	*ctx*AB (%)	*hly*A (%)	*tcp* (%)
1	2007	1.RAYAGADA 2. PURI	O1 (n = 10)	100	100	40	50	60	100	100	90	100	90
	2007		NON-O1/ NON-O139 (n = 4)	75	50	25	50	25	100	25	0	75	25
2	2009	(1) RAYAGADA (2) PURI (3) KENDRAPARA	O1 (n = 14)	100	42.8	35.7	42.8	42.8	100	100	100	100	50
3	2010	(1) RAYAGADA (2) PURI	O1 (n = 3)	100	33.3	33.3	33.3	66.6	100	100	66.6	100	66.6
	2010		NON-O1/ NON-O139 (n = 4)	75	0	0	0	25	100	0	25	100	100
4	2011	(1) RAYAGADA (2) GAJAPATI	O1 (n = 1)	100	0	0	0	100	100	100	100	100	100
	2011		NON-O1/ NON-O139 (n = 4)	100	100	0	75	100	100	50	25	100	50
5	2012	(1) RAYAGADA (2) KALAHANDI	O1 (n = 14)	100	100	100	100	42.8	100	100	85.7	100	57.1
	2012		NON-O1/ NON-O139 (n = 2)	100	100	100	100	0	100	0	100	100	100
6	2013	(1) KORAPUT (2) RAYAGADA	O1 (n = 3)	100	100	100	100	0	100	100	100	100	66.6
7	2016	1. BALASORE	O1 (n = 1)	100	100	100	100	100	100	100	100	100	100
8	2017	1. RAYAGADA	NON-O1/ NON-O139 (n = 13)	100	46	0	15.3	30.7	100	7.6	46.2	76.9	0
9	2018	1. RAYAGADA	O1 (n = 4)	100	100	100	100	100	100	100	100	100	100
	2018		NON-O1/ NON-O139 (n = 5)	100	80	20	20	60	100	60	100	80	20
10	2019	1. RAYAGADA	O1 (n = 1)	100	100	100	100	100	100	100	100	100	100
	2019		NON-O1/ NON-O139 (n = 2)	100	100	0	50	0	100	0	50	100	0

**Table 2:** Annual antibiogram profiles of *Vibrio cholerae* O1 and non-O1/non-O139 strains isolated from environmental sources in different districts of Odisha: 2007–2019

Serial No.	YEAR	AREA	SEROGROUP (n)	G	Cf	Nx	At	Of	Do	A	C	Fr	Co	S	Na	N	T	PROFILES	
1	2007	(1) RAYAGADA (2) PURI	O1 (n = 10)	90	80	0	90	10	0	100	100	100	100	100	100	100	0	G,Cf,At,A,C,Fr,Co,S,Na,N	
	2007	(1) RAYAGADA (2) PURI	NON-O1/NON-O139 (n = 4)	50	0	0	50	0	0	100	100	100	50	50	50	75	0	G,At,A,C,Fr,Co,S,Na,N	
2	2009	(1) RAYAGADA (2) PURI (3) KENDRAPARA	O1 (n = 14)	57	57	0	50	14	0	100	21	100	21	100	36	100	21	G,Cf,At,A,Fr,S,N	
3	2010	(1) RAYAGADA (2) PURI	O1 (n = 3)	67	66.6	0	67	0	0	100	0	100	33.3	100	0	100	66.6	.G,Cf,At,A,Fr,S,N,T	
	2010	(1) RAYAGADA (2) PURI	NON-O1/NON-O139 (n = 4)	75	50	0	50	25	0	100	25	100	25	100	25	100	25	G,Cf,At,A,Fr,S,N	
4	2011	(1) RAYAGADA (2) GAJAPATI	O1 (n = 1)	0	0	0	0	0	0	100	100	100	100	100	100	0	100	A,C,Fr,Co,S,Na,T	
	2011	(1) RAYAGADA (2) GAJAPATI	NON-O1/NON-O139 (n = 4)	0	0	0	0	0	0	100	0	100	75	100	100	75	25	A,Fr,Co,S,Na,N	
5	2012	(1) RAYAGADA (2) KALAHANDI	O1 (n = 14)	85.7	64.2	0	64.2	14.3	0	100	50	100	57.1	100	100	92.9	7.1	G,Cf,At,A,C,Fr,Co,S,Na,N	
	2012	(1) RAYAGADA (2) KALAHANDI	NON-O1/NON-O139 (n = 2)	50	50	0	100	50	0	100	0	100	50	100	100	100	0	G,Cf,At,Of,A,Fr,Co,S,Na,N	
6	2013	(1) KORAPUT (2) RAYAGADA	O1 (n = 3)	33.3	0	0	33.3	0	0	100	100	100	100	100	100	33	0	A,C,Fr,Co,S,Na,	
7	2016	1. BALASORE	O1 (n = 1)	0	0	0	100	0	0	100	0	100	100	100	100	0	0	At,A,Fr,Co,S,Na	
8	2017	1. RAYAGADA	NON-O1/NON-O139 (n = 13)	61.5	15.4	0	15.4	7.7	0	100	0	100	30.8	100	69.2	100	7.7	G,A,Fr,S,Na,N	
9	2018	1. RAYAGADA	O1 (n = 4)	0	0	0	100	0	0	100	0	100	100	100	100	0	0	AT,A,Fr,Co,S,Na	
	2018	1. RAYAGADA	NON-O1/NON-O139 (n = 5)	0	0	0	0	0	0	100	0	100	0	100	20	100	0	A,Fr,S,N	
10	2019	1. RAYAGADA	O1 (n = 1)	0	0	0	0	0	0	100	0	100	100	100	100	100	0	A,Fr,Co,S,Na,N	
	2019	1. RAYAGADA	NON-O1/NON-O139 (n = 2)	0	0	0	50	0	0	100	0	50	0	100	0	100	0	At,A,Fr,S,N	

[Gentamicin (G), ciprofloxacin (Cf), norfloxacin (Nx), azithromycin (At), ofloxacin (Of), doxycycline (Do), ampicillin (A), chloramphenicol (C), furazolidone (Fr), cotrimoxazole (Co), streptomycin (S), nalidixic acid (Na), neomycin (N) and tetracycline (T)]

**Fig. 3:**
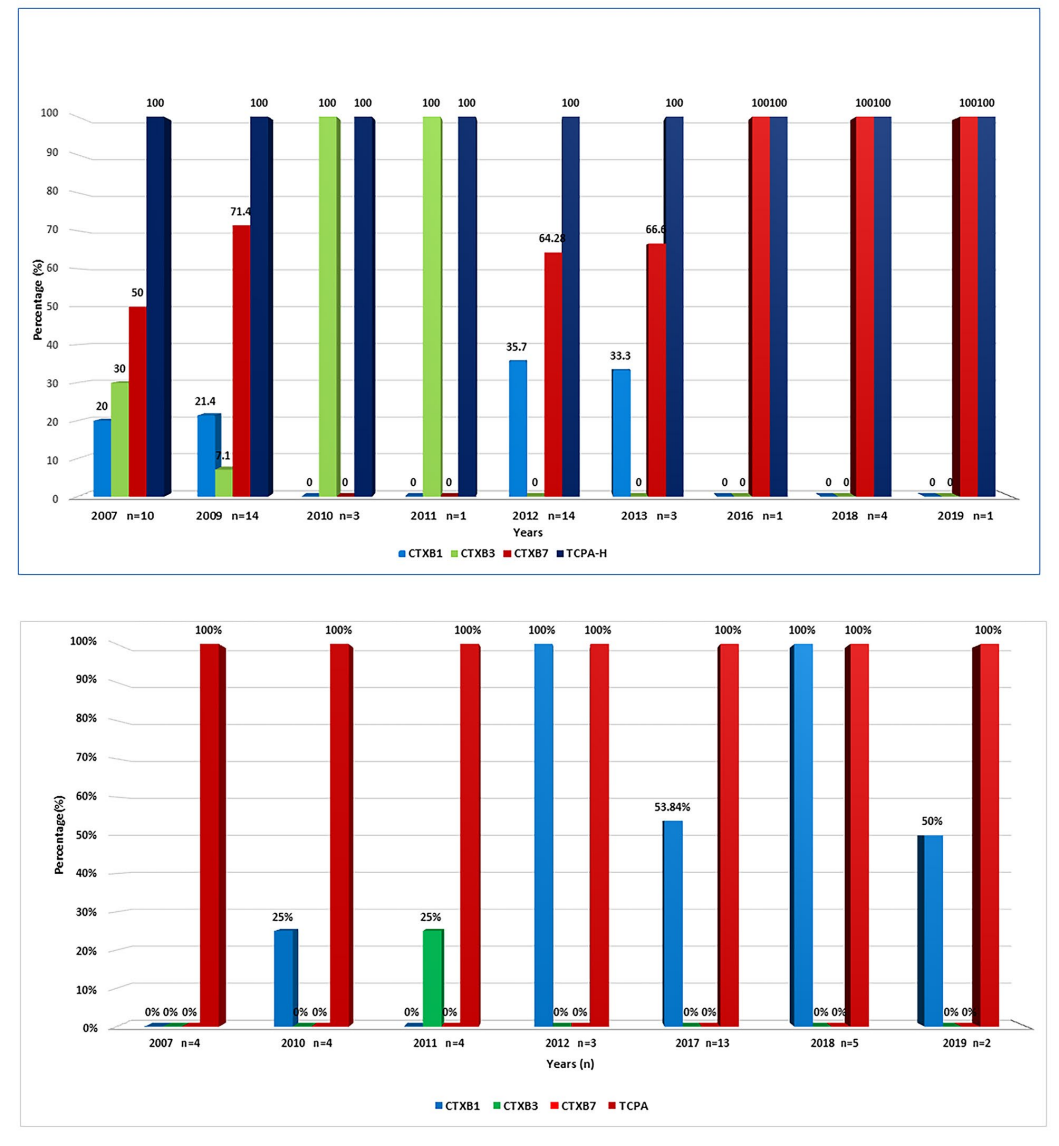
(a): Prevalence of different virulence genes like *ctx*B and *tcp*A genes in *Vibrio cholerae* O1 strains from environmental water isolates. (b): Prevalence of different virulence genes like *ctx*B and *tcp*A genes in *Vibrio cholerae* non-O1/non-O139 strains from environmental water isolates

**Fig. 4:**
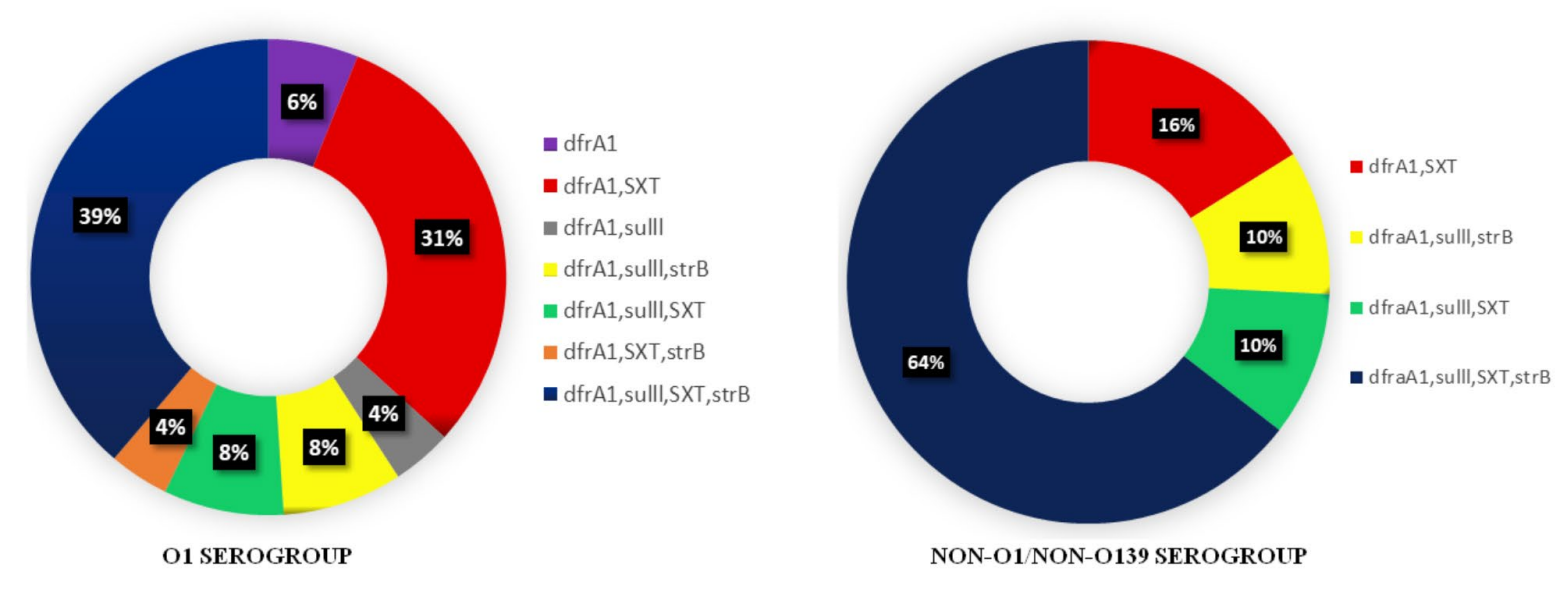
Distribution of antibiotic resistant genes in *Vibrio cholerae* O1 and non-O1/non-O139 strains represented in doughnut chart

**Fig. 5:**
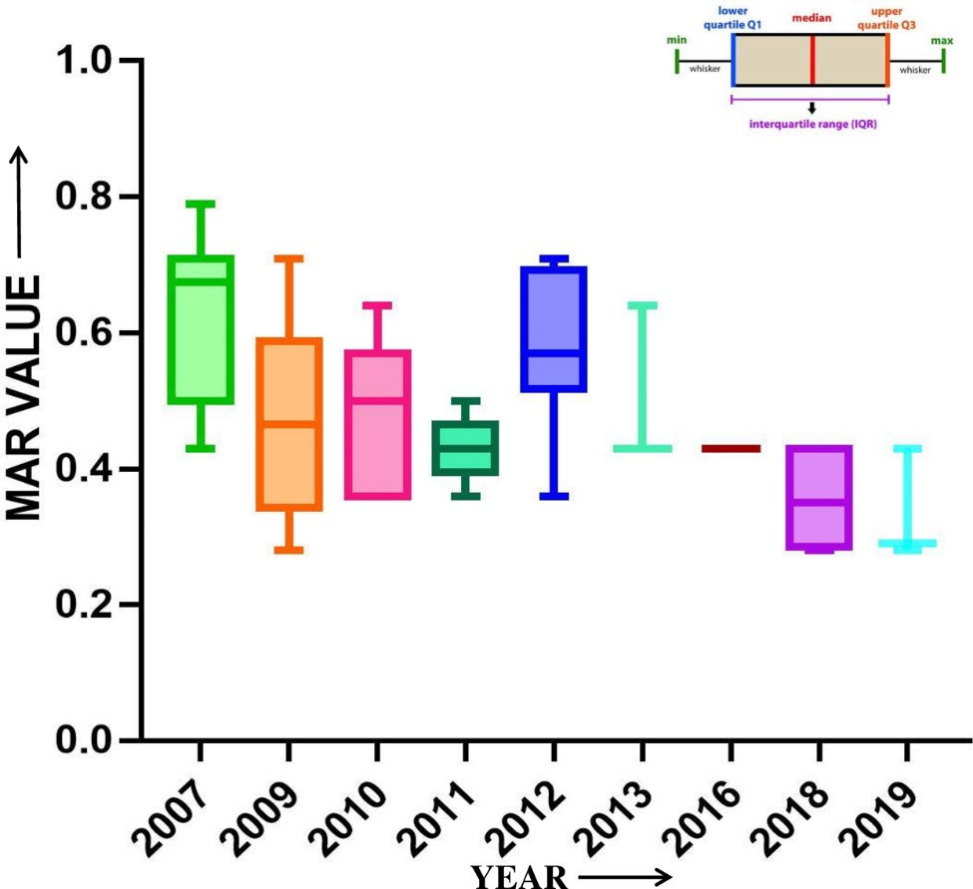
Multiple antibiotic resistance (MAR) Index of *Vibrio cholerae* O1 isolates represented in Box whisker plot

**Fig. 6:**
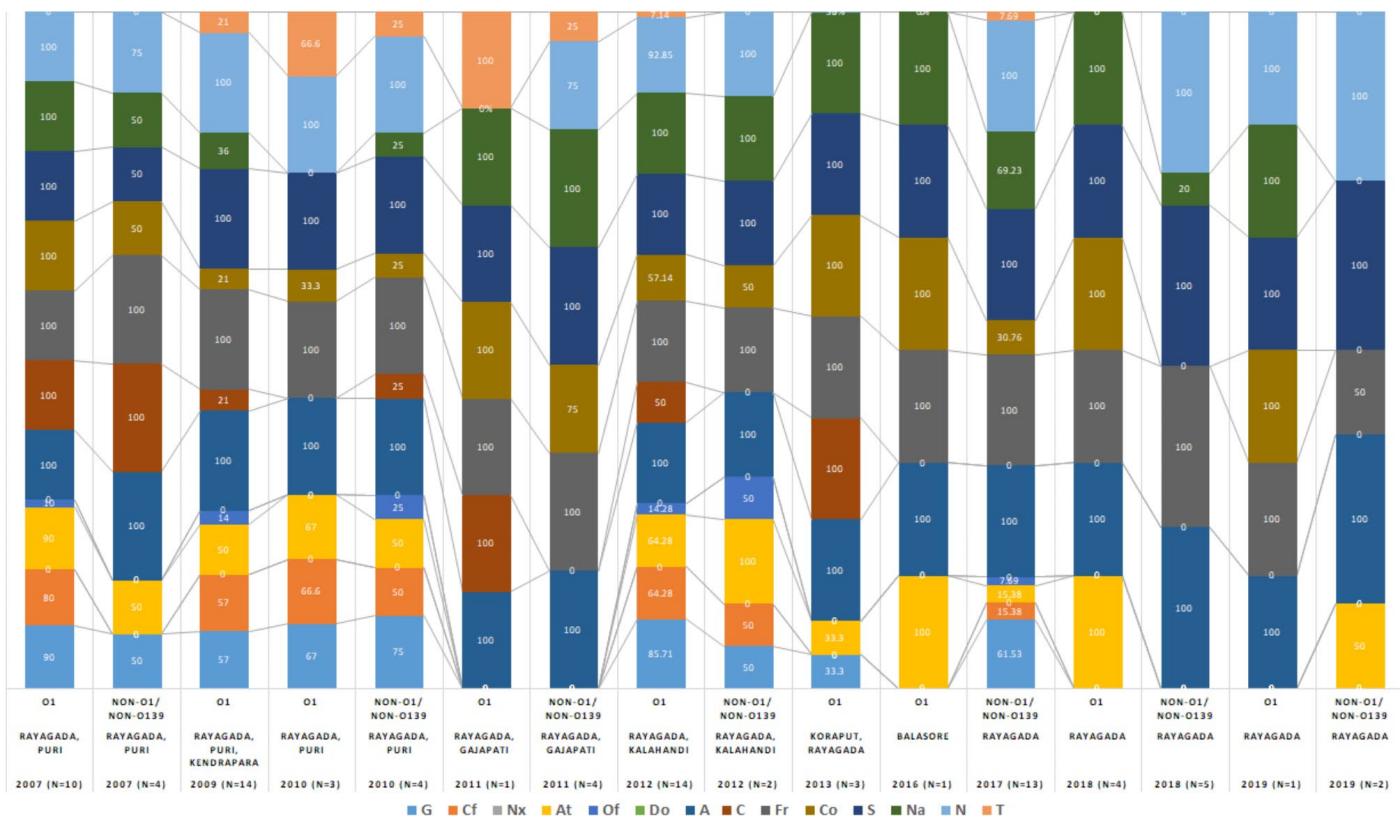
Antibiogram profiles of *Vibrio cholerae* O1 and non-O1/non-O139 strains represented in stacked column chart [gentamicin (G), ciprofloxacin (Cf), norfloxacin (Nx), azithromycin (At), ofloxacin (Of), doxycycline (Do), ampicillin (A), chloramphenicol (C), furazolidone (Fr), cotrimoxazole (Co), streptomycin (S), nalidixic acid (Na), neomycin (N) and tetracycline (T)]

**Fig. 7:**
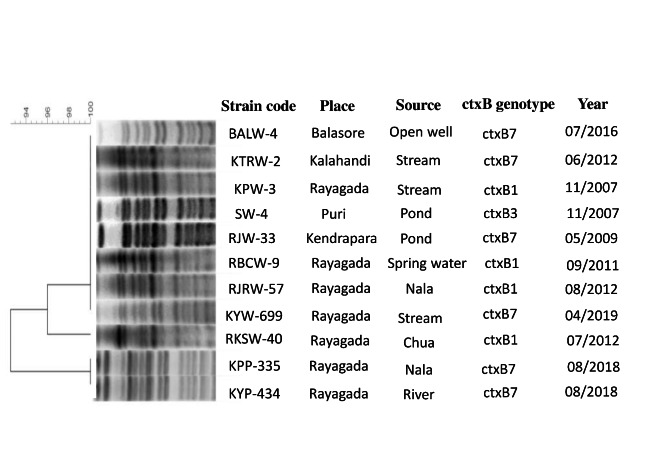
PFGE analysis of *Not*I digested *Vibrio cholerae* O1 strains isolated from environmental sources in different districts of Odisha: 2007–2019

## Data Availability

All data are available within the manuscript.
